# Improved Surface Quality and Microstructure Regulation in High Power Fiber Laser Cutting of Stainless Steel Grid Plates

**DOI:** 10.3390/ma17235959

**Published:** 2024-12-05

**Authors:** Linjiang Xu, Chunming Wang, Fei Yan, Zhuangxi Hu, Wei Zhang

**Affiliations:** 1 School of Materials Science and Engineering, Huazhong University of Science and Technology, Wuhan 430074, China; m202271048@hust.edu.cn (L.X.); hzx7851423@163.com (Z.H.); zhangweid2022@hust.edu.cn (W.Z.); 2 School of Automotive Engineering, Wuhan University of Technology, Wuhan 430070, China; 3 Ningbo Xiangming Laser Technology Co., Ltd., Ningbo 315000, China

**Keywords:** laser cutting, 304 stainless steel, gap overlapping cutting, section analysis, microstructure

## Abstract

In order to disintegrate nuclear fuel rods in the grid connection structure, a 10 kW fiber laser was used to cut a stainless steel simulation component with four layers of 3 mm thick plates and 12 mm gaps. The slit width is regarded as an important indicator to evaluate the cutting quality of the four-layer stainless steel plate. The results showed that good laser cutting quality can be successfully achieved under the proper process parameters. The widths of the cut seams of the four layers of grating after cutting were 1.25, 1.65, 1.80, and 1.92 mm. As the auxiliary gas pressure decreased layer by layer, the metal melting pool for the first two plates was mainly destroyed by the auxiliary gas. The cutting quality was good, and the slit area was mainly austenite with the presence of some ferrite. The third- and fourth-layer plates almost had no gas flow to assist blowing off, so the cut surface was an uneven melting pit, the cutting quality was poor, and the cut seam area ferrite content was higher than the upper plate cut seam area. At the same time, due to the lack of airflow cooling of the bottom plate, high laser energy, and long heating time, grain coarsening occurred, while grain deformation and a large number of dislocations existed. It can provide process support and technical guidance for the disintegration of nuclear fuel rods.

## 1. Introduction

In recent years, the application prospect of nuclear power has been becoming more and more extensive at home and abroad [1]. Nuclear fuel rods are an important source of energy for nuclear power [2]. The disintegration and recovery of nuclear fuel rods have been an extremely important part of the nuclear fuel cycle system [3]. At present, the common method of fuel rod decomposition is mechanical shearing [4]. However, it easily gives rise to tool wear during the cutting process, subsequently resulting in the difficulty of tool replacement and other problems. Therefore, it is very difficult for it to be widely used in the field of atomic energy [5]. Water jet cutting produces liquid waste easily [6]; plasma cutting cannot be applied in the radiation environment of the hot room and destroys the atmosphere structure of the hot room [7]. The essence of laser cutting is to use the thermal energy of a high-energy laser beam to disintegrate the material [8]. Compared with other methods, laser cutting has obvious advantages in thermal chambers, such as a narrow slit, a small heat-affected zone, a large cutting depth to width ratio, and high cutting efficiency [9,10]. At the same time, laser processing does not need to worry about tool wear, mechanical vibration, and other issues [11,12], which makes it suitable for application in the disintegration of nuclear power fuel rod recycling [13,14].

Koji et al. [15] conducted a 30 kW fiber laser cutting of simulated steel components and thick steel plates in the field of nuclear power generation and obtained a good cutting quality. The results proved the superiority of laser cutting in the field of nuclear decommissioning and the prospect of development. Shi et al. [16] conducted 30 kW fiber laser cutting on 40 mm thick 304 stainless steel for a four-factor five-level orthogonal test and explored the influence of each process parameter on the cutting section index law. The results showed that the slit width affected the focus position, auxiliary gas pressure, laser power, and cutting speed. Liao et al. [17] applied a 10 kW fiber laser to cut a 20 mm thick 20CrNiMo steel plate under the condition of unassisted gas blowing. They confirmed the feasibility of laser cutting, but the dripping of molten metal under gravity led to a poor cut quality. Nathan et al. [18] performed a 3 kW fiber laser cutting on seven layers of 0.35 mm thick M250-35A grade electrical steel sheet laminates. It was found that when seven layers were cut in stacks, adhesion occurred between the bottom steel sheets and the cut quality was poor. Kwan et al. [19] carried out underwater laser cutting of 50 mm thick STS304L specimens and explored the decommissioning and disintegration of nuclear power plant structures. They found that the best cutting surface quality was obtained at a cutting speed of 30 mm/min with a focus position of 30 mm and laser power of 9 kW. Duan et al. [20] applied aerodynamics to investigate the gas flow field in the kerf and found that the distributions of the gas velocity and pressure were nonlinear and strongly dependent on the pre-cutting geometry and the gas jet boundary conditions.

Among the above studies, considerable research has been conducted by scholars on the process of the laser cutting of carbon and stainless steel in the nuclear field and the relationship between cutting quality and its process parameters [21,22]. However, current research is mostly focused on simulating the laser cutting of flat plates in a nuclear environment, and few studies have been reported on the laser cutting of more complex shaped components, especially for the application of the disintegration of nuclear fuel rod components [23]. This paper took the stainless steel grid plate in nuclear fuel rods as the research object. Considering that the disintegration process was in the hot room environment, the grid plate was connected to the ends of the complex fuel rod assembly [24]. It is not possible to remove and cut each single-layer plate sequentially. Therefore, a method for cutting four-layer stainless steel gap-stacked plates without removal is necessary, as is the study of the impact of different process parameters on component cutting [25]. For multi-layer board gap-stacking cutting, due to the non-contact between the different layers of the boards, heat conduction is blocked, and the temperature difference of the slit between multi-layer boards is quite large [26]. At the same time, due to the auxiliary gas and because the laser can only pass through the upper plate slit to the lower plate, the bottom plate is subjected to a small auxiliary gas pressure and a nonuniform laser, leading to bottom plate cutting difficulties and a poor cutting quality [27]. Research on the cutting process window and section quality of four-layer stainless steel plate cutting can provide technical support for the disintegration of nuclear fuel rods.

## 2. Materials and Methods

**Figure 1:**
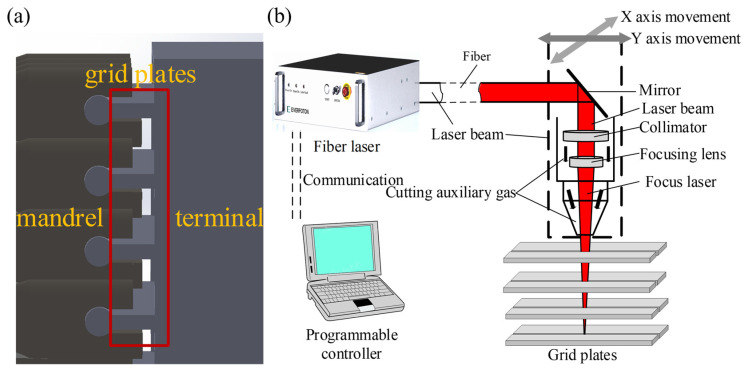
Experimental devices: (**a**) the structure of the multilayer grid plate; (**b**) cutting experimental setup.

The structure of the key connecting structure in the nuclear fuel rods is shown in Figure 1a. It consists of four layers of 3 mm thick stainless steel. The gap between the stainless steel plates is 12 mm, and the mandrels and ends are connected on both sides of the plates. In this paper, four layers of 3 mm thick 304 stainless steel plates were used to simulate the combined grating assembly for the laser cutting test. The 304 stainless steel used in the experiment was a room temperature austenitic structure. The microstructure for the equiaxial crystalline austenite grains was interspersed with some striated ferrite, and its chemical composition (wt.%) is shown in Table 1 [28].

The experimental laser was the YMM-10000 fiber laser system of Guozhi Laser (Dongguan, China). The main parameters were as follows: a central wavelength of 1080 ± 10 nm, a maximum output laser power of 10 kW, a modulation frequency of 50 kHz, and a core diameter of the output fiber of 100 μm. The laser cutting head was the Precitec’s Pro Cutter 15 kW laser cutting head (Draisstraße 1, Gaggenau, Germany). The motion mechanism used was the three-dimensional CNC laser cutting machine of Huagong Farley (Wuhan, China), which controlled the motion of the laser cutting head through the CNC machine to complete the cutting experiment with fast cutting speed and high stability. The CNC machine can move at a maximum speed of 80 m/min in the X/Y axis and 60 m/min in the Z. The cutting process used N_2_ as an auxiliary gas to remove stainless steel slag, and the pressure of the auxiliary gas can be up to 24 bar.

The evaluation index of cutting quality was selected from three aspects, including the cutting width, slag height, and the roughness of the cutting surface. According to these measurement indices, different measuring equipment was used to observe the kerf width, slag height, and the kerf section morphology of the four-layer stainless steel gap-laminated plate. The morphology after cutting was analyzed by scanning the processed photos with a scanner. The kerf width and slag height of the fourth-layer stainless steel plate were obtained using a vernier caliper and a spiral micrometer for theoretical analysis. The morphology and roughness of the cutting section were analyzed by confocal analysis. Since the application scenario is the disintegration of grids for nuclear spent fuel rods, the core metric considered is the width of the cut seam. The quality of the slits affects the subsequent steps in the disintegration of the fuel rods. First of all, the width of the grating itself determines that the slit width should be as small as possible; the width of the grating intervals requires that the slag hanging height should be much smaller than the interval width, so as to avoid the two layers sticking to each other. In the end, it is necessary to ensure a smooth separation of the lower end from the mandrel for post-dissolution recycling.

**Figure 2:**
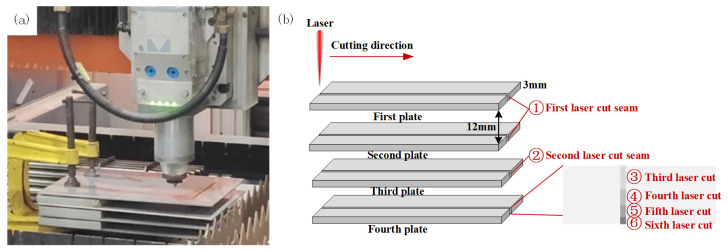
Stack cutting of four-layer stainless steel plate with gaps: (**a**) experimental platform for stacked cutting; (**b**) illustration of multiple laser cuts for stacked cutting.

The cutting experimental platform is shown in Figure 2a. In the process of cutting four layers of stainless steel gap laminates using a fiber laser metal cutting machine, the coaxial auxiliary blowing method was adopted to ensure less oxidation and a smaller heat-affected zone. The auxiliary inert gas used in this work was ordinary nitrogen. The shielding device adopted a supersonic nozzle with an outlet diameter of 1.5 mm. As shown in Figure 2b, the laser cutting path was unidirectional linear cutting. For the four-layer stainless steel plate, the first three layers used two lasers to obtain better quality slits based on the previous experiments. For the fourth layer of the stainless steel plate, unidirectional multi-channel laser linear cutting was used, and the number of cutting times was six. The length of the slit was 100 mm. In order to prevent the thermal effect of the adjacent slits, the distance between the two adjacent slits was set to 50 mm. Before the experiment, the surface of the plate was wiped with anhydrous ethanol to remove oil and other pollutants, subsequently ensuring the uniformity of the absorption rate of the experiment.

In this work, the impact of different factors on the cutting of the plate was investigated using a single-factor experimental method. Process test parameters are shown in Table 2.

## 3. Results and Discussion

### 3.1. Influence of Process Parameters on Cutting Quality

**Figure 3:**
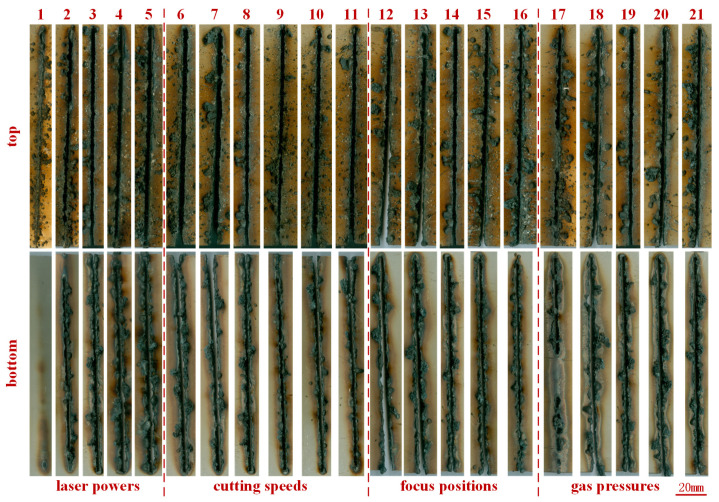
Macroscopic morphology of the cut seam with different parameters.

According to the designed process parameters, the simulated sample was cut. The cutting morphology of the fourth layer plate is shown in Figure 3. Because the auxiliary gas pressure of the fourth layer was the smallest when cut by gap superposition, the laser needed to cut the fourth layer through the first three layers of slits, resulting in the worst cutting quality.

Measured values of kerf width, slag height, and roughness after the cutting of the fourth layer plate are shown in Table 3 below.

**Figure 4:**
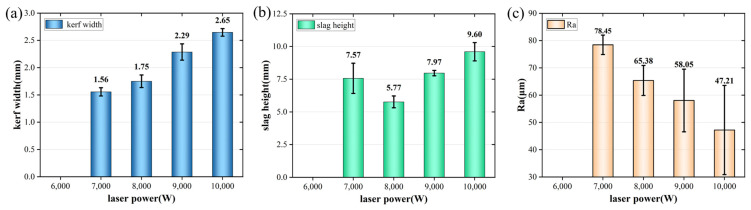
Cutting results of the fourth-layer plate under different laser powers: (**a**) slit width; (**b**) slag hanging height; (**c**) cut surface roughness.

In order to investigate the influence of laser power on the cutting results, the power was set to 6000~10,000 W, and the remaining parameters were a cutting speed of 600 mm/min, a first laser focus position of −14 mm, a subsequent laser focus position of −30 mm, an auxiliary gas pressure of 17 bar, a gas nozzle diameter of 1.5 mm, and a cutting height of 1 mm. As shown in Figure 3, the fourth layer cannot be cut through at 6000 W, and then the cutting was unstable at 7000 W. It was observed that there was a local adhesion in some groups of experiments. As shown in Figure 4, with the increase in laser power, the slit width increased significantly, and there was also an upward trend in the height of slag hanging after complete penetration. This is because as the laser power increases, the heat input increases. A large amount of metal is melted, and the auxiliary gas is not blown out in time. The molten metal can drip slowly, and the final slit width and slag height increase. There was a downward trend in surface roughness. Considering that laser power had a significant effect on the slit width, a laser power of 8000 W was finally selected.

**Figure 5:**
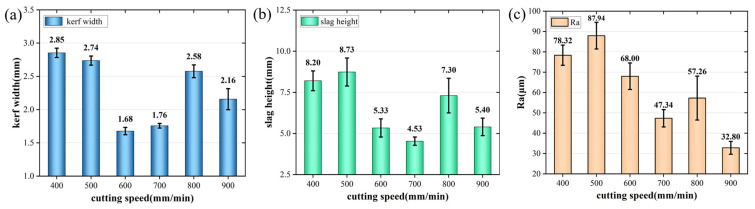
Cutting results of the fourth-layer plate at different cutting speeds: (**a**) width of cutting seam; (**b**) height of slag hanging; (**c**) roughness of cut surface.

The cutting results were analyzed at different cutting speeds. The parameters were set to 400~900 mm/min. The remaining parameters were a laser power of 8000 W, a first laser focus position of −14 mm, a subsequent laser position of −30 mm, and an auxiliary gas pressure of 17 bar. As shown in Figure 3, the four-layer steel plate can be cut through under each group of parameters. As shown in Figure 5, the mechanism of cutting speed is similar to that of the laser power. With the increase in cutting speed and the heat input decreased, the molten metal reduced, and the slit became narrower. However, when the cutting speed increased to a certain level, the metals melted, subsequently resulting in a wider slit. The end result appeared to be a smaller kerf width at medium cutting speeds. The slag height varied similarly to the kerf width, with lower slag heights at moderate cutting speeds. The surface roughness decreased with the increase in cutting speed. Based on this, a cutting speed of 600 mm/min was selected.

**Figure 6:**
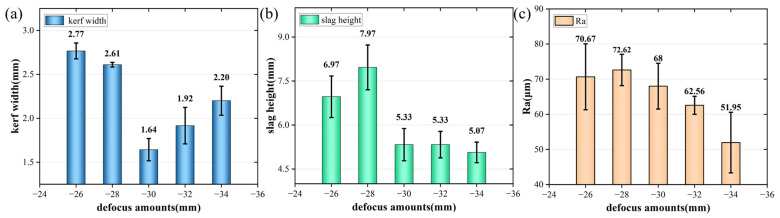
Experimental results of the fourth-layer plate at different focus positions. (**a**) Width of cutting slit; (**b**) height of slag hanging; (**c**) roughness of cut surface.

The experimental results at different focus positions are shown in Figure 6. The main parameters were as follows: a first laser focus position of −10~−18 mm, and the subsequent five laser focus positions of −26~−34 mm. The remaining parameters were a laser power of 8000 W, a cutting speed of 600 mm/min, and an auxiliary gas pressure of 17 bar. As shown in Figure 3, four layers of stainless steel plates can be cut through under different parameters. As shown in Figure 6, when the focus position was high, the spot diameter of the fourth layer of the stainless steel plate was very large. Because the heat input was sufficient at this time, more base metal was melted, resulting in a wider slit. When the focus position was reduced to a certain extent, the laser defocusing amount and surface spot diameter became larger, resulting in a wider slit of the first three layers. This led to a large heat input in the fourth layer. The slit of the fourth layer also increased. Finally, the slit width decreased first and then increased. The slag height became high with the increase in the focus position. However, the slag height was not much different with the decrease in the focus position. The surface roughness decreased slowly with the decrease in the focus position. Finally, −14 mm and −30 mm were selected as the first focus position and subsequent laser focus position, respectively.

**Figure 7:**
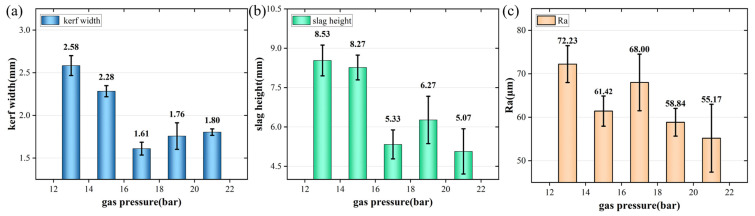
Cutting results of the fourth layer plate with different auxiliary gas pressures. (**a**) Width of cutting slit; (**b**) height of slag hanging; (**c**) roughness of cut surface.

The experimental results under different auxiliary gas pressures were shown in Figure 7. The experimental parameters were set to 13~21 bar, and the remaining parameters were a laser power of 8000 W, a cutting speed of 600 mm/min, a first laser focus position of −14 mm, and a subsequent laser position of −30 mm. As shown in Figure 3, when the auxiliary gas pressure was 13 bar, the fourth layer could not be cut through. However, when the gas pressure reached 15 bar, the fourth layer could be cut through. As shown in Figure 7, when the auxiliary gas pressure was small, the kerf width and the slag height were both large due to the difficulty of blowing off the molten metal in time. When the auxiliary gas pressure reached a certain standard, the kerf width and slag height decreased to a certain range. The surface roughness decreased slowly with the increase in gas pressure. Because of this reason, the auxiliary gas pressure was selected to be 17 bar.

The final process window was ascertained, including a laser power of 8000 W, a cutting speed of 600 mm/min, a first laser focus position of −14 mm, a subsequent laser focus position of −30 mm, and an auxiliary gas pressure of 17 bar.

### 3.2. Cutting Morphology of Four-Layer Stainless Steel Plate

**Figure 8:**
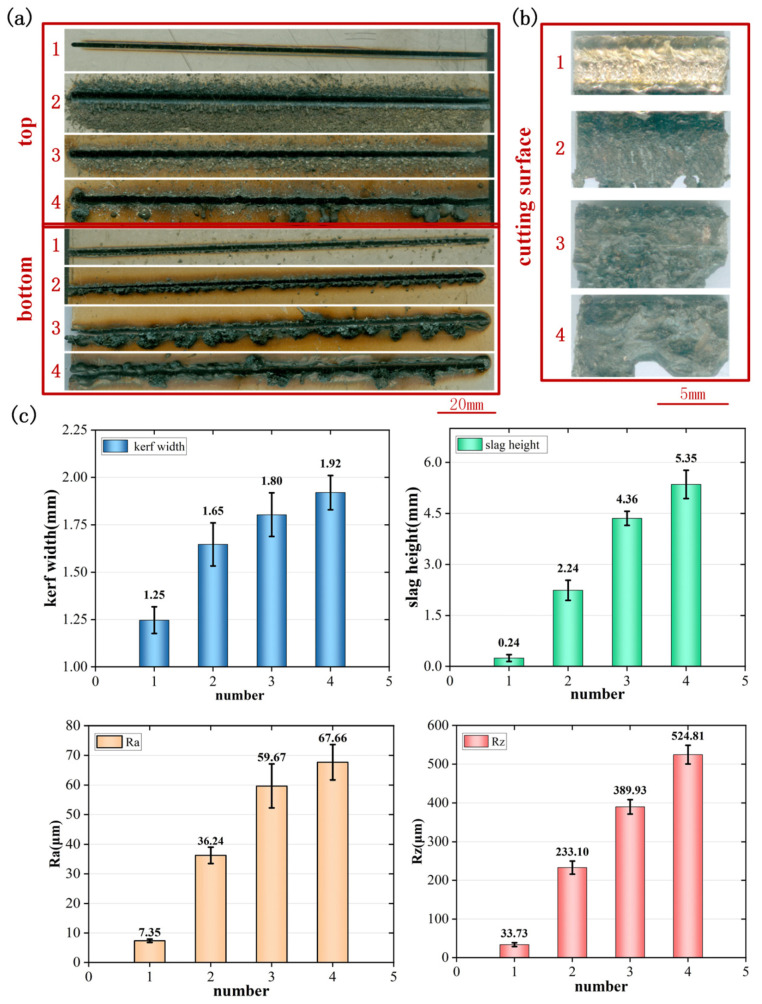
Cutting results of four-layer stainless steel plate: (**a**) top and bottom morphology of the cut seam; (**b**) side morphology of the cut seam; (**c**) cut seam analysis.

The front and back surfaces of the simulated sample under the optimal process are shown in Figure 8a. It can be seen that the first two layers of slits were straight, and the slit width did not change significantly. The fourth-layer slit was relatively straight, the third-layer slit width changed slightly, and the fourth-layer slit width changed more obviously. The slag on the front and back of the first-layer plate was reduced. In the front of the second-layer plate, there was uniform and fine slag dispersed on both sides of the slit. This was because the waste slag of the first-layer plate slit moved downward through the high-pressure auxiliary airflow and dispersed on the front of the second-layer plate. Due to a decrease in the auxiliary gas pressure at the position of the second plate, some slag could not be completely blown off. Therefore, there was a small amount of slag at the lower end of the slit, and most of the molten metal was blown down to the front of the third plate to form fine and uniform slag. There were uneven, large droplets of slag on the back of the third-layer plate and the surface of the fourth-layer plate. The reason was that the auxiliary gas pressure at the position of the three or four layers was very small, which made the slag unable to be blown away, and the slag mainly drops under the action of gravity. Therefore, there were three layers of slag on the front of the four layers and uneven large blocks of slag on the back of the three or four layers.

As shown in Figure 8b, the laser cutting section of the four-layer plate was obviously different. The cutting section of the first-layer plate was flat and bright. The cutting surface of the second layer was relatively rough, and there were drag marks on the surface. The oxidation of the cutting surface became black and gray, and the cutting quality was poor. The cutting surface of the third and fourth layers was rough, and the surface was an irregular melting pit. The cutting surface was obviously oxidized and blackened, and the cutting quality was poor. Due to sufficient heat input from the laser cutting of the first-layer plate, the plate was melted enough, and the auxiliary gas was enough to blow off the slag. The section showed bright luster and was relatively smooth and of the best quality. The pressure of the auxiliary gas at the second layer began to decrease significantly; the pressure decreased, and a vortex was formed, which was not easy for blowing off the slag. The molten metal dripped under the action of the weak auxiliary gas pressure and gravity. At the same time, due to the small nitrogen pressure, it was difficult to form a nitrogen atmosphere environment. The section was oxidized and discolored, so the cutting section was a gray-black droplet section. The auxiliary gas pressure of the third and fourth plates was very small, and there was almost no blowing left and right. The molten metal droplets destroyed the molten pool droplets under the action of gravity, forming an uneven melting pit-like section. The section was oxidized and blackened, and the cutting quality was poor.

As shown in Figure 8c, the final four-layer plate slit widths were 1.25, 1.65, 1.80, and 1.92 mm, respectively, and the slag heights were 0.24, 2.24, 4.36, and 5.35 mm, respectively. Cutting surface roughness values were 7.35, 36.24, 59.67, and 67.66 μm, while in the traditional laser cutting of a 3 mm thick 304 stainless steel plate cutting surface roughness is only 4.32 μm. Gap-stacking cutting a four-layer plate when the bottom plate roughness is much higher than the traditional laser cutting surface; the width of the cut seam and the height of the slag in the first four layers of the board increased in sequence, and the section roughness of the four-layer plate also increased. The cutting width, slag height, and surface roughness of the first, second, and third layers were quite different. The cutting width, slag height, and surface roughness of the third and fourth layers were relatively close. This was because the removal mechanism of the third and fourth layers was similar, which was obviously different from the first and second layers.

### 3.3. Slag Removal at Different Auxiliary Gas Pressure

The process model of laser cutting the simulated sample was described as a two-dimensional plane symmetrical gas flow field model. The model was used to simulate the pressure change process of the auxiliary gas in the melting and cutting process. The process optimization experiment and the establishment of the slag force model provided a regular summary and simulation verification. In this model, the slit was the area where nitrogen interacted with the steel wall. The auxiliary gas was ejected from the nozzle and passed through the slits with widths of 1.4, 1.5, 2.0, and 2.4 mm. The distance between the nozzle and the upper surface of the first layer of the stainless steel plate was 1.0 mm. The areas established by the model were all fluid areas. The surface adjacent to the solid area and the fluid area was set as the wall. The nozzle was simplified into a conical nozzle, and the outlet diameter of the nozzle was set to 1.5 mm.

The two-dimensional modeling of the flow field was carried out in Design Modeler. The boundary conditions were set as follows: The boundary condition of the nozzle inlet surface was set as the pressure inlet. The nozzle wall surface, the slit side, and the upper and lower surfaces of the four-layer stainless steel plate were set as the wall. The nozzle middle section and the slit middle section were set as the symmetrical surface. The nozzle and the first layer of the stainless steel plate interval area boundary, the interval area boundary between each layer of plate, and the fourth layer of the stainless steel plate bottom air area boundary and other surfaces were uniformly set as the pressure outlet. The inlet temperature and outlet temperature were set to 298 K. The inner wall of the slit and the inner wall of the nozzle were set as the boundary conditions of the non-slip wall. In the calculation process, the SIMPLE algorithm was used in this model, and the least squares cell-based spatial discretization method was used. The pressure control mode was second order. The turbulent kinetic energy control method was first order upwind.

**Figure 9:**
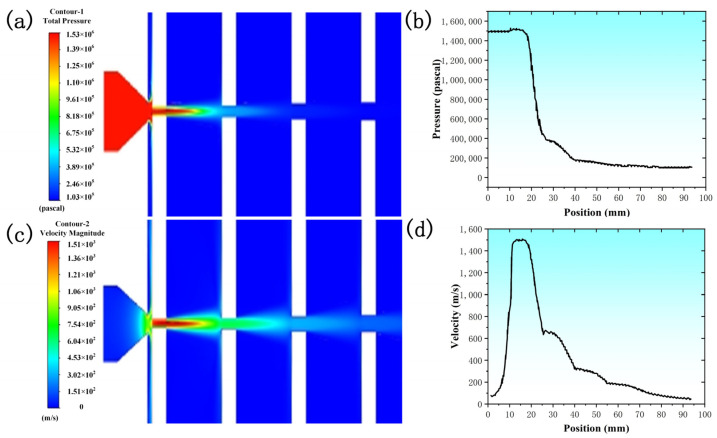
Variation of auxiliary gas pressure and flow rate in a four-layer stainless steel plate: (**a**) auxiliary gas pressure cloud; (**b**) auxiliary gas pressure and distance from nozzle variation; (**c**) auxiliary gas flow rate cloud; (**d**) auxiliary gas flow rate and distance from nozzle variation.

The initial auxiliary gas pressure was set to 15 bar, and the Fluent software (Fluent 2022 R1)was used to simulate the airflow. The auxiliary gas pressure cloud diagram at different positions of the four-layer stainless steel plate is shown in Figure 9a. The white position was the stainless steel plate, the blue position was the gap, and the pressure at the second plate decreased sharply. The change of the auxiliary gas pressure and the distance from the nozzle are shown in Figure 9b. The pressure began to decrease sharply near 17 mm. Finally, the air pressure at the surface of the four-layer plate was 15, 4.2, 1.7, and 1.3 bar, respectively. The auxiliary gas flow velocity contours at different positions are shown in Figure 9c. The gas flow velocity of the first layer was the largest and then changed layer by layer. The variation of the auxiliary gas flow rate and the distance from the nozzle is shown in Figure 9d. The gas flow rate curve had an inflection point at the position of each layer of the stainless steel plate, and the flow rate changed significantly. Finally, the gas velocity at the midpoint of the four-layer plate was 862, 584, 279, and 165 m/s.

**Figure 10:**
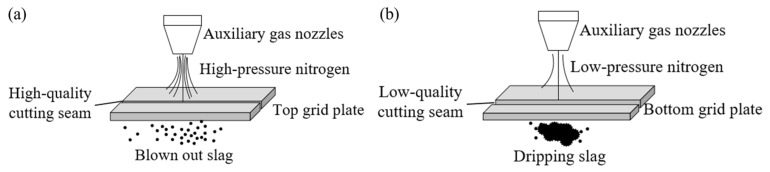
Slag removal methods under different auxiliary gas pressures. (**a**) Schematic diagram of slag removal using high-pressure auxiliary gas blowing; (**b**) schematic diagram of slag dropping using low-pressure auxiliary gas.

According to the simulated results, it can be seen that due to the change of auxiliary gas pressure, the size of auxiliary gas pressure at the position of the first plate was almost constant. The auxiliary gas pressure at the position of the second plate was sharply reduced, and the auxiliary gas pressure at the position of the third and fourth plates had almost no blowing-off effect. As shown in Figure 10a, the auxiliary gas pressure of the top plate was enough to blow away the slag, so the experimental results of the cross-section showed a bright-colored luster and were smoother and of the best quality. The second layer of auxiliary gas pressure began to reduce, making it significantly difficult to blow off all the molten metal, so the formation of droplets of metal attached to the gravity and the role of auxiliary gas dropped. As a result, the cut surface was rougher, resulting in the cut surface being oxidized to a blackish gray. As shown in Figure 10b, in the bottom grating there was almost no gas flow to assist the blowing effect, so the cut surface was uneven and melting pit-like and obviously oxidized to black.

### 3.4. Microstructure of Four-Layer Stainless Steel Plate Cut Seam

**Figure 11:**
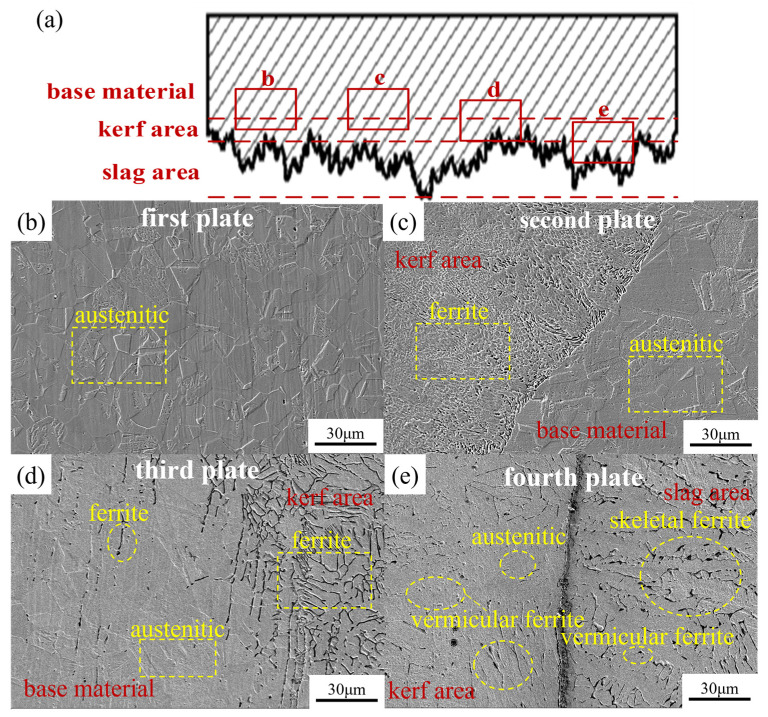
SEM images of a four-layer 304 stainless steel plate in the cut seam. (**a**) Schematic diagram of the cross-section of the slit; (**b**) first-layer plate; (**c**) second-layer plate; (**d**) third-layer plate; (**e**) fourth-layer plate.

Figure 11 shows the SEM image of the simulated sample. Different heat inputs and different auxiliary airflow pressures of the four-layer plate affected the microstructure of the kerf and the slag hanging area. The sample material was 304 stainless steel, in which the equivalent of chromium nickel was 18.52 and 9.59. The chromium–nickel equivalent ratio was 1.93. When the nickel–chromium equivalent ratio was between 1.5 and 2.0, it was in FA solidification mode, and the initial precipitated phase was ferrite. Before the end of solidification, some austenite structures were formed by a peritectic–eutectic reaction, which existed at the boundary of δ ferrite [29].

As shown in Figure 11a, due to a large enough heat input during the cutting of the first layer of the stainless steel plate, the molten metal was blown off by a large enough auxiliary gas pressure. The molten metal was in a rapid non-equilibrium solidification process, but the laser influence area was small, and the action time was short. Finally, the microstructure of the slit area was still dominated by the same austenite as the base material.

Figure 11b is the junction of the kerf melting zone of the second layer of the stainless steel plate and the substrate-affected zone. The heat-affected zone of the base material was mainly austenite, and a small amount of ferrite appeared between the austenite cells. During the thermal cycle in the heat-affected zone, the austenite phase transformation into ferrite was relatively slow, so the number of ferrites formed was not large. In the kerf area, due to the large heat input, the temperature rose rapidly. Under the large auxiliary gas pressure of the second layer plate, the cooling rate was moderate, and the austenite phase transformation was transformed into ferrite, so there was a large amount of ferrite in the kerf area.

Figure 11c is the junction of the kerf melting zone and the substrate-affected zone of the third layer of the stainless steel plate. The heat-affected zone of the base material was still dominated by austenite, and there was more ferrite between the austenite cells. In the thermal cycle process of the heat-affected zone, the laser action made the temperature rise fast. Because the auxiliary gas pressure was too small, the gas took away less heat, and the cooling rate was slow, which led to the faster transformation of austenite to ferrite, and the final formation of ferrite was more than that of the second-layer plate. The difference between the kerf area and the second-layer plate was that the diffusion of austenite during the phase transformation was limited. When the diffusion distance decreased, it was easier to carry out the phase transformation in a closely arranged lath shape, which resulted in the residual ferrite becoming a lath shape. As for the bottom plate, high laser density and low auxiliary gas pressure resulted in a high kerf temperature and a slow cooling rate. Lamellar and skeletal ferrites easily form because the atoms have enough time to diffuse during the slow cooling process. The carbon in the austenite gradually diffuses, and the iron atoms rearrange to form ferrite. The ferrite precipitates in lamellar or skeleton-like forms, and the remaining carbon is enriched in other phases, such as carburization.

Figure 11d is the junction of the kerf melting zone and the slag hanging zone of the fourth-layer stainless steel plate. As shown in the figure, the kerf melting zone was mainly composed of ferrite, and there were skeleton ferrite and vermicular ferrite. This was due to the long laser action time at the fourth-plate cut; the temperature raised quickly, the molten pool temperature was high, and the cut had a slow cooling rate under the small auxiliary gas pressure. Finally, the austenite was transformed into ferrite in large quantities. The slag zone was the same as the kerf melting zone. The temperature rose fast, resulting in slow cooling, and a large amount of austenite was transformed into ferrite. The slag hanging area was mainly composed of skeleton ferrite, and more vermicular ferrite appeared. The high energy input of the laser and the fast temperature rise caused the austenite to deform, producing a large number of dislocations. During cooling, these dislocations provided additional nucleation sites, and the ferrite grew in a specific direction, culminating in a worm-like shape.

Laser power, cutting speed, and focus position affect the laser energy density, while auxiliary gas pressure affects the molten metal blowing speed and cooling time. When the laser energy density is larger and the cooling time is longer, the solidification time of the molten metal pool becomes longer, the growth time of dendrites becomes longer, and grain coarsening occurs. At the same time, ferrite-generating elements such as chromium remaining in the ferrite continue to enrich. Austenite-generating elements such as nickel and carbon continue to deplete. Until the process stabilizes the ferrite at a diffusion-restricted lower temperature, the end result is an increase in the ferrite content of the bottom plate [30].

Ferrite is less strong and tough compared to austenite. When subjected to external forces, the ferrite region may become a weak point and is prone to cracking, thus reducing the integrity of the component. At the same time, ferrite and austenite have different electrode potentials, which can form microcells and accelerate the corrosion process under the corrosive environment. It can lead to defects such as corrosion pits on the surface of the component, reducing the effective bearing area of the component and affecting its integrity [31].

**Figure 12:**
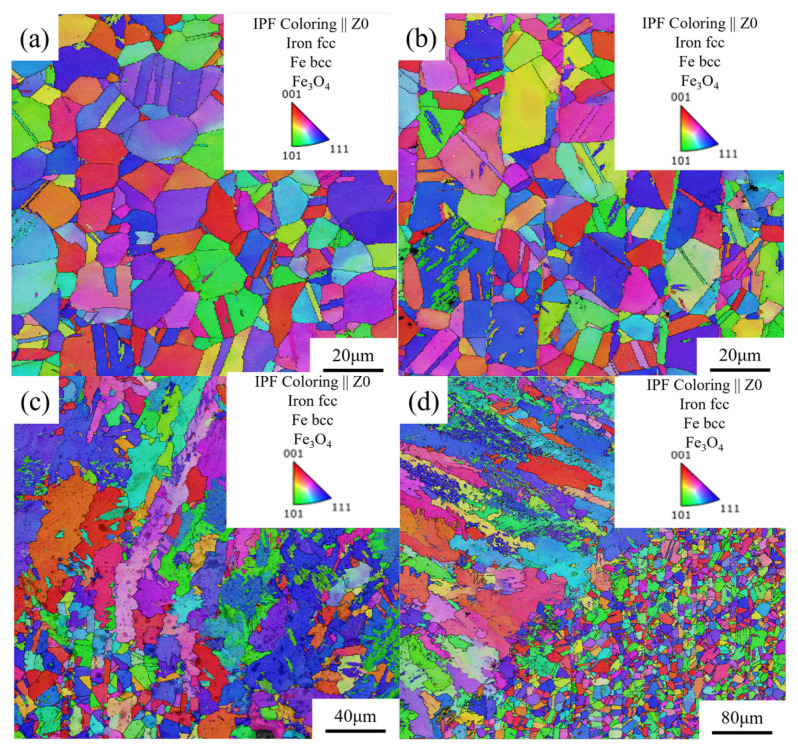
EBSD–BC + IPF + GB image of the four-layer 304 stainless steel plate at the cut seam. (**a**) First-layer plate; (**b**) second-layer plate; (**c**) third-layer plate; (**d**) fourth-layer plate.

Figure 12 is the EBSD-BC, IPF, GB image of the simulated sample. As shown in Figure 12, the EBSD-GB diagrams at the four-layer plate slit show that the original austenite grain boundaries of the four-layer plate slit are still continuous. However, the grain boundaries of the first and second plates are straight, and the grain boundaries in the region of the cut seam of the third and fourth plates are curved, which have been changed during the laser thermal action. As shown in Figure 12a,b, the grain orientation at the slit of the first and second plates was random, and there was no special polarity orientation behavior. At the same time, no obvious abnormal grain coarsening phenomena were found in the first and second laminates, showing good microstructure stability. After statistics, the grain sizes were 9.74 and 10.72 μm, respectively, which were basically the same as the grain size of the base metal. As shown in Figure 12c,d, there was an obvious grain coarsening phenomenon near the laser heat source, which meant that the heat of the laser cutting of the third and fourth layers of stainless steel reached the temperature of stainless steel phase transformation, and then coarsening occurred, which can clearly distinguish the heat-affected zone from the base metal grains. After statistics, the grain sizes were 53.23 and 85.02 μm, respectively. The microstructure after coarsening still showed random grain orientation behavior, and no special polar orientation behavior occurred.

Coarsening of the grain reduces the strength and toughness of the material. The area of grain boundaries between coarse grains is relatively small, and when subjected to external forces, dislocation motion is more likely to pass through the grain boundaries, making the material more prone to plastic deformation or even fracture, which is not conducive to maintaining the integrity of the shape and performance of the component [32].

**Figure 13:**
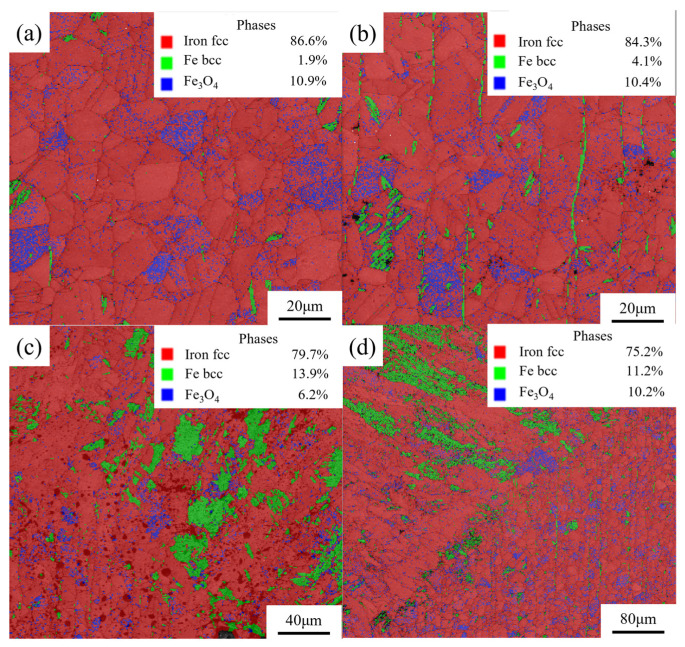
EBSD–BC + Ph image of the four-layer 304 stainless steel plate at the cut seam. (**a**) First-layer plate; (**b**) second-layer plate; (**c**) third-layer plate; (**d**) fourth-layer plate.

Figure 13 shows the EBSD-BC and Ph images of the simulated sample. As shown in Figure 13, there were some phases with various structures, such as fcc and bcc, at the kerf, and there was oxidation to form Fe_3_O_4_ at the kerf position. The amount of ferrite produced by austenite transformation varies from laminate to laminate. The content of bcc at the kerf of the four layers of the stainless steel plates was 4.1%, 1.9%, 13.9%, and 11.2%, respectively. As shown in Figure 13a,b, the austenite transformation occurred in the kerf area, and a small amount of ferrite was formed. The distribution of ferrite was not obvious, and the content was below 5%. As shown in Figure 13c,d, due to a large heat input, small pressure of the auxiliary gas, and a long heating time, the obvious austenite transformation occurred in the kerf area, and a large amount of ferrite was generated. A large amount of ferrite was distributed in the kerf heat-affected area. There was obvious regularity, and the content was above 10%.

**Figure 14:**
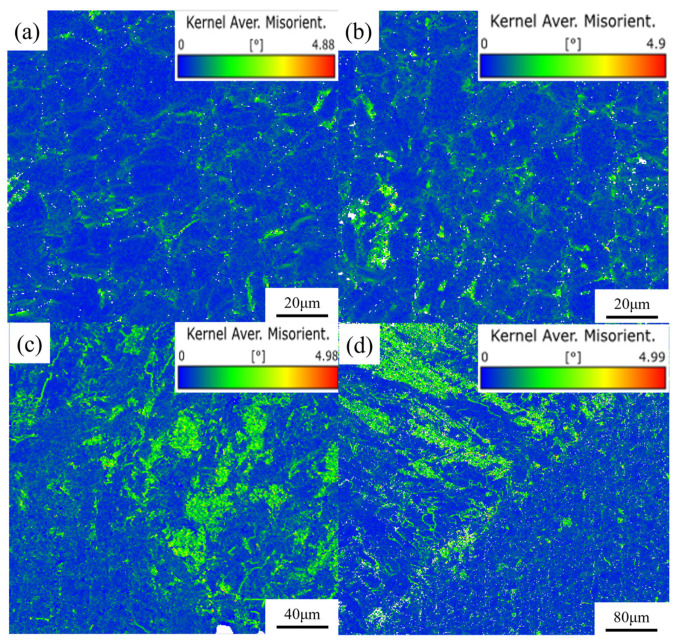
EBSD–KAM image of the four-layer 304 stainless steel plate at the cut seam. (**a**) First-layer plate; (**b**) second-layer plate; (**c**) third-layer plate; (**d**) fourth-layer plate.

Figure 14 is the EBSD-KAM image of the kerf of the simulated sample. As shown in Figure 14a,b, no local stress concentration was found during laser cutting, which further confirmed that the first and second laminates had good cutting formation. While the three or four layers of the plate were heated for a long time by the laser, the heat was transmitted to the surrounding area, making the heat-affected zone of the material larger. The material produced a large expansion due to heat, and a large residual stress was formed after cooling. As shown in Figure 14c,d, a significant local misorientation (KAM) was observed in the melting region, which reflected the degree of homogenization of plastic deformation. The higher the value, the greater the degree of plastic deformation or the higher the defect density. Combined with the grain boundary images of Figure 12c,d, the following analysis can be made: under the stress deformation caused by thermal accumulation, a large number of dislocations were accumulated inside this heat-affected region. Then, the dislocation density gradually increased, and the dislocation wall was formed by dislocations intertwining with each other, and the dislocation wall further developed into a subgranular boundary, constituting a subcrystal, and dynamic recovery occurred. When the stress in the heat-affected zone accumulated to a certain critical value, the subgrain boundary continued to absorb dislocations, and dynamic recrystallization occurred. The subgrain boundary gradually transformed into a large-angle grain boundary. It can be seen that there were a large number of dislocations inside the deformed grains.

### 3.5. Microhardness of Slit Cross-Section

**Figure 15:**
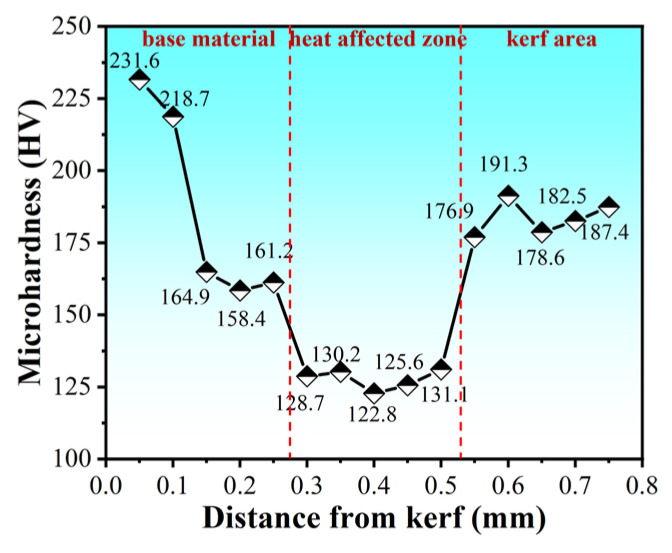
Distribution of microhardness in the cross-section of the fourth laminate cutout.

Figure 15 shows the microhardness distribution curve of the 304 stainless steel slit cross-section from the slit edge to the substrate direction. As can be seen from Figure 15, the hardness of the melting zone, the heat-affected zone, and the substrate of the three parts varies. The 304 stainless steel substrate hardness is 187.4 HV. The melting zone edge hardness reaches 231.6 HV. The edge of the microstructure is dense, and even partially oxidized, part of the hardness reaches 821.7 HV. The middle of the melting zone hardness reaches 158.4 HV, due to the heat flow cycle leading to inconsistent dendrite directions. The microstructure was not dense enough, and the corresponding hardness is lower than the hardness of the matrix and the edge of the melting zone. The heat-affected zone hardness is 122.8 HV, significantly lower than the hardness of the melting zone and the matrix. Although the heat-affected zone recrystallization may cause a fine crystallization effect, the temperature of the heat-affected zone closer to the melting zone boundary is higher. The longer stagnation time at high temperature is conducive to the growth of grains. The austenite grains tend to coarsen on account of the influence of thermal cycling [33], reducing the hardness of the heat-affected zone.

## 4. Conclusions

In this study, multiple 3 mm thick 304 stainless steel plates were used as materials to study the gap-laminated cutting process of four-layer 304 stainless steel plates and find the cutting process window. Combined with changes in the auxiliary gas pressure and the flow rate of four-layer plates, the kerfs of the four-layer stainless steel plates after cutting were discussed from the perspectives of section morphology and microstructure. The following conclusions were obtained:

(1) Four layers of 304 stainless steel plate gap stack were successfully cut. The main process window for better cutting quality was as follows: a laser power of 8000 W, a cutting speed of 600 mm/min, an auxiliary gas pressure of 17 bar, and a laser focus position of −14 mm on the first layer, followed by five laser focus positions of −30 mm.
(2) In a four-layer stainless steel plate, the slit width and slag height increased progressively from the top layer to the bottom. The cutting quality of the top layer was optimal, while the bottom layer exhibited an uneven, pitted melting surface with significantly poorer cutting quality.
(3) Auxiliary gas pressure and flow rate decreased dramatically at the second plate position. The mechanism of molten metal removal in the third and fourth layers of the plate was mainly slag dripping under gravity, resulting in significant changes in the cut surface morphology, slit width, and slag height compared with the first two layers.
(4) The first layer of the stainless steel plate seam area was mainly the same as the austenite-based parent material. Ferrite appeared in the second layer of the plate seam area. A large amount of slate ferrite appeared in the third and fourth layers of the plate cut seam. The hanging slag area of the skeleton ferrite was dominated by worm-like ferrites.
(5) There was obvious grain coarsening in the seam area of the third and fourth plates. Moreover, in the melting region, significant local orientation differences appeared. Heat accumulation led to stress deformation in the melting region.

## Figures and Tables

**Table 1:** Chemical composition list of base metals.

Element	S	P	C	Si	Mn	Ni	Cr	Fe
Content/(wt.%)	0.004	0.02	0.06	0.86	1.84	9.59	18.52	Bal

**Table 2:** Four-layer stainless steel plate gap-lamination cutting process parameter design table.

Number	Laser Power (W)	Cutting Speed (mm/min)	Defocus Amount (mm)	Auxiliary Gas Pressure (bar)
1	6000	600	−14/−30	17
2	7000	600	−14/−30	17
3	8000	600	−14/−30	17
4	9000	600	−14/−30	17
5	10,000	600	−14/−30	17
6	8000	400	−14/−30	17
7	8000	500	−14/−30	17
8	8000	600	−14/−30	17
9	8000	700	−14/−30	17
10	8000	800	−14/−30	17
11	8000	900	−14/−30	17
12	8000	600	−10/−26	17
13	8000	600	−12/−28	17
14	8000	600	−14/−30	17
15	8000	600	−16/−32	17
16	8000	600	−18/−34	17
17	8000	600	−14/−30	13
18	8000	600	−14/−30	15
19	8000	600	−14/−30	17
20	8000	600	−14/−30	19
21	8000	600	−14/−30	21

**Table 3:** Measurement table for the fourth-ply cut seam indicator.

Number	Kerf Width (mm)	Slag Height (mm)	Ra (μm)
1	Not cutting through		
2	1.56	7.57	78.45
3	1.75	5.77	65.38
4	2.29	7.97	58.05
5	2.65	9.60	47.21
6	2.85	8.20	78.33
7	2.74	8.73	87.94
8	1.68	5.33	68.00
9	1.76	4.53	47.34
10	2.58	7.30	57.26
11	2.16	5.40	32.80
12	2.77	6.97	70.67
13	2.61	7.97	72.62
14	1.64	5.33	68.00
15	1.92	5.33	62.56
16	2.20	5.07	51.95
17	2.58	8.53	72.23
18	2.28	8.27	61.42
19	1.61	5.33	68.00
20	1.76	6.27	58.84
21	1.80	5.07	55.17

## Data Availability

The original contributions presented in this study are included in the article. Further inquiries can be directed to the corresponding authors.
